# Genome-wide association study provides genetic insights into natural variation in watermelon rind thickness and single fruit weight

**DOI:** 10.3389/fpls.2022.1074145

**Published:** 2022-12-06

**Authors:** Chengsheng Gong, Xuqiang Lu, Hongju Zhu, Muhammad Anees, Nan He, Wenge Liu

**Affiliations:** ^1^ Zhengzhou Fruit Research Institute, Chinese Academy of Agricultural Sciences, Zhengzhou, China; ^2^ Jiangsu Key Laboratory for Horticultural Crop Genetic Improvement, Institute of Vegetable Crops, Jiangsu Academy of Agricultural Sciences, Nanjing, China

**Keywords:** watermelon, GWAS, rind thickness, single fruit weight, transcriptome

## Abstract

Rind thickness and fruit weight are agronomic traits closely related to quality and yield, which have attracted much attention from consumers and breeders. However, the genetic mechanism of these two traits is still not well understood in natural populations. In this study, rind thickness and single fruit weight in 151 watermelon accessions were determined in 2019 and 2020, and genome-wide association analysis was performed by integrating phenotypic and genotype data. Abundant phenotypic variation was found in the test population, and the watermelon with thinner rind thickness tended to have smaller fruit weights. Five significant SNPs were closely associated with rind thickness on chromosome 2 by Genome-wide association study (GWAS), i.e., 32344170, 32321308, 32304738, 32328501, and 32311192. And there were 21 genes were annotated in the candidate interval, most notably, *Cla97C02G044160* belonged to the MADS family, and part of the genes in this family played an important role in regulating organ size, further analysis of gene structure, gene expression level, and phylogenetic tree showed that *Cla97C02G044160* was a candidate gene for regulating target traits. In conclusion, our study provides molecular insights into the natural variation of watermelon rind thickness and single fruit weight, meanwhile, providing data support for molecular marker-assisted breeding.

## Introduction

As one of the five most popular consumed fresh fruits, watermelon [*Citrullus lanatus* (Thunb.) Matsum. &Nakai var. lanatus] is widely cultivated in the world and brought considerable economic benefits (Guo et al., 2013; Gong et al., 2021b). With the increasing market demand for high-quality watermelons, research on important phenotypic traits such as fruit shape, flesh color, fruit sugar content, and metabolic traits has been deepened (Ren et al., 2017; Dou et al., 2018; Zhang et al., 2020; Gong et al., 2021a). Rind thickness and single fruit weight are two important agronomic traits that have attracted much attention in the market. Specifically, small-fruit watermelon with thinner rind tends to have better flavor and is an important component of high-quality watermelon in the market, while large-fruit watermelon with thicker rind thickness contributes to the increase of watermelon yield and transportation.

The research on the genetic basis of important agronomic traits is helpful to accelerate the modern breeding process (Ren et al., 2014). Forward genetics carries out genetic basis research based on phenotypic variation, and the relevant research is often carried out by genetic map, Bulked Segregant Analysis (BSA), and Genome-wide association study (GWAS). Advances have been made in gene mapping of single fruit weight and rind thickness through forwarding genetics, and key QTL has been obtained in cucumber, melon, watermelon, and other Cucurbitaceae crops (Pan et al., 2020). For instance, Zhong et al. (2017) obtained QTL loci linked to 12 important agronomic traits such as rind thickness based on the construction of a high-density genetic map of pumpkin, providing theoretical support for mining candidate genes and molecular marker breeding. Research on QTL linked to fruit thickness in wax gourds has also made progress (Liu et al., 2018). In watermelon, (Wi et al. 2012) obtained QTLs linked to fruit size, rind thickness, and other traits through different genetic populations; Ren et al. (2014) realized the main QTL loci for 12 agronomic traits such as fruit length and sugar content by integrating four genetic maps, including QTLs related to rind thickness and fruit weight, i.e., *RTH2-1*, *RTH5*, *RTH6*, *FWT2-1*, *FWT2-2*, *FWT3*, *FWT5-1*, and *FWT5-2*. In addition, forward genetics combined with transcriptome data is helpful for further mining and identifying key candidate genes that regulate target traits. Compared with genetic map and BSA, GWAS could be used to analyze the association between polymorphic nucleic acids and traits in a constructed population, and quickly identify target loci and possible regulatory genes associated with traits (Hamblin et al., 2011; Yano et al., 2016).

At present, important progress has been made in the research on the physiological and genetic basis of plant fruit weight and rind thickness, and some key candidate genes have been identified. The size of normal organs is usually affected by the coordination of cell proliferation and cell expansion (Horiguchi et al., 2006). Genes can regulate the weight of fruit, rind thickness, and the size of other traits by regulating the content of auxin, ethylene, cytokinin, and other plant hormones (Le et al., 2001; Wilmoth et al., 2005; Di Marzo et al., 2020). In addition, transcription factors (TFs) can also play an important role in regulating the size of plant tissues and organs, notably, the MADS family belongs to TFs and plays an important role in regulating the size of tissues and organs. For instance, in Arabidopsis, the TF of MADS gene *GORDITA* (GOA) has the function of regulating fruit growth, and mutation or reduced expression of this gene results in larger fruit (Prasad et al., 2010); *SEEDSTICK* regulating cytokinin levels and other genes to controls fruit weight of *Arabidopsis* (Di Marzo et al., 2020); In addition, rind thickness and fruit size have been extensively studied in tomato, and the genes such as *SlARF7* were considered to be key candidate genes related to regulating tomato rind thickness (De Jong et al., 2009). Furthermore, the construction of metabolic regulatory networks based on gene expression levels is helpful to elucidate the genetic basis of important agronomic traits (Umer et al., 2020). However, the current research on the key candidate genes regulating target traits is still far from enough.

Research on the genetic basis of single fruit weight and rind thickness in watermelon can help meet the practical needs of production, progress has been made in the study of rind thickness and fruit weight, while the understanding of the variation and genetic basis of these two traits in the natural population is still insufficient. In the present work, variation analysis and correlation analysis were carried out after the single fruit weight and rind thickness were measured of 151 watermelon accessions in 2019 and 2020. Combined with high-quality SNP data, GWAS of the target traits was performed and significant SNPs were obtained. Further, combine bioinformatics analysis and transcriptome data mining to identify key candidate genes that regulate target traits. In conclusion, our study provides molecular insights into the natural variation of rind thickness and single fruit weight and contributes to marker-assisted breeding and new cultivars selection.

## Materials and methods

### Plant cultivation and management

151 watermelon accessions belong to cultivated cultivars (*C. lanatus cultivar* and *C. lanatus landrace*), with bright flesh color to facilitate the identification of rind thickness. These accessions were randomly selected from previous studies with resequencing data (Guo et al., 2019; Gong et al., 2022), and were collected in the National Mid-term Genebank for Watermelon and Melon, Zhengzhou Fruit Research Institute, Chinese Academy of Agricultural Sciences. The plants were planted in Xinxiang (35.30°N, 113.88° E) in 2019 and Zhongmu (34.72°N, 113.98 E) in 2020, respectively. The rind of 14 watermelon accessions with different thicknesses (grey background in **Table S1**) was sampled at the maturity period for qRT-PCR (Quantitative real-time polymerase chain reaction, qRT-PCR). In addition, two watermelon cultivars ‘97103’ and ‘203Z’ with different single fruit weights (‘97103’:3.2 kg, ‘203Z’:2.0 kg) and rind thicknesses (‘97103’:1.1 cm, ‘203Z’:0.5 cm) were mainly used for transcriptome determination and planted in Sanya, Hainan in the winter of 2021. And the plants were transplanted through seedling raising and according to the local climate conditions. Twin-vine pruning was used, and only one watermelon fruit per plant was retained. The main temperature and humidity in the greenhouse were as follows: during the daytime, the temperature was kept between 25°C to 35°C, and the humidity was 55%-70%, while at nighttime, the temperature was often above 15°C, and the humidity was maintained at 75% to 80%. Alternatively, irrigation, watering, and fertilization were managed according to the actual needs of watermelon growth and were managed in the same way. The cultivars were harvested according to their ripening characteristics, and then, the single fruit weight and rind thickness were measured.

### Determination of phenotypic data and sampling

For the 151 watermelon accessions, three mature watermelons with good growth were randomly selected from each cultivar for the determination of target traits, and the phenotype data after calculating the average value were used for further analysis. The electronic scale was used to measure the weight of the fruit, and after the watermelon fruit was cut lengthwise, the thickness of the rind was measured with a ruler. Watermelon accessions ‘97103’ and ‘203Z’ were used to perform transcriptome sequencing, and 14 watermelon accessions were used to qRT-PCR, the rind of watermelon without exocarp was sampled at mature period, and the watermelon was cut vertically into 1 cm thick slices along the largest diameter, and cuboid pericarp of about 1 cm in length and width was taken from the four vertical directions and mixed as a sample to be tested. Three watermelons of the same growth were individually sampled as triplicate biological replicates.

### GWAS and candidate gene identification

In the previous study, the linkage disequilibrium (LD) decay rates of the *C. lanatus* cultivar and *C. lanatus* landrace were relatively low (Guo et al, 2019), showing a high degree of domestication, and eliminating the influence of wild-type cultivars on genotype analysis due to differences in genetic background to a certain extent. The resequencing data of 126 watermelon accessions were obtained from (Guo et al. 2019) (accession number WM in **Table S1**), and the other 25 were obtained from Gong et al. (2022) (accession number R in **Table S1**). The method of gene library construction and resequencing was consistent with a previous study (Guo et al., 2019). TruSeq Nano DNA High Throughput Library Prep Kit was used to construct about 400 bp sequences. Sequencing was performed on the Illumina HiSeq X or HiSeq 2000 platform at Berry Genomics (Beijing, China). And the watermelon genome 97103 v2 (http://cucurbitgenomics.org/organism/21) was used as the reference genome for the related analysis, and QQ plots were plotted to predict the reliability of the model. The Manhattan plot and QQ plot were drawn by R software. The SNP data of mutation detection were filtered according to the secondary allele frequency (MAF: 0.05) and locus integrity (INT: 0.8), and the high-quality SNP was obtained for further analysis. For GWAS, a Factored Spectrally Transformed linear mixed model (FaST-LMM) method was used for relevant analysis, and QQ plots were plotted to predict the reliability of the model, and EMMA eXpedited(EMMAX) and Linear Model are used to verify the reliability of the location results. The Manhattan plot and QQ plot were drawn by R software. And the modified Bonferroni correction was used to determine two significance thresholds, i.e., -log10 (P) 0.1/Ne (Ne = effective SNP number) and -log10 (P) 0.01/Ne0.01/SNPs and 0.1/SNPs, respectively. Criteria for identifying significant SNPs: when the SNP exceeded the top threshold in the Manhattan plot, it was considered to be highly significantly associated with the target trait and SNP. When the lower threshold line was exceeded, the correlation between the two was considered significant. When located below the threshold line, SNPs that are close to the threshold line and show a continuous distribution in a small interval were also considered may be associated with the target trait.

According to the analysis of LD decay in natural populations in previous studies (Guo et al., 2019), we took the interval of 100 kb upstream and downstream of the significant SNP locus as the candidate interval. Furthermore, combined gene annotation information, previous research reports, and transcriptome data to screen possible candidate genes. Gene sequence information was obtained from the online website of the cucurbitaceous crop genome (http://www.cucurbitgenomics.org/).

### Transcriptome sequencing and qRT-PCR

The rind samples of ‘97103’ and ‘203Z’ with three biological repeats were ground into powder in liquid nitrogen and then used for RNA extraction. RNA extraction was performed according to the instructions of the TRlzol Reagent (Life Technologies, California, USA). Qualified high-quality RNA was used for the next research, and NEBNext Ultra RNA Library Prep Kit for Illumina (NEB, E7530) and NEBNext Multiplex Oligos for Illumina (NEB, E7500) was used for cDNA library construction. The cDNA library was sequenced at an Illumina HiSeq™ sequencing platform. The original sequencing data were filtered to obtain clean data, which were compared with the watermelon genome 97103 v2, and further quality control was performed to obtain high-quality data. FPKM values (number of exon kilobases per million segments) were used to estimate gene expression levels (Florea et al., 2013). When the fold change of FPKM value between the two cultivars exceeded 1.8, the gene was defined as a differential gene. According to previous research reports, the genes related to seed expansion and hormones in watermelons were counted, and the heat map was drawn based on the expression level of Origin software (V 8.0.724).

Fourteen watermelon cultivars were set with three biological replicates. The powdered watermelon rind samples were extracted with the RNA isolation kit (Huayueyang Biotechnologies, China) for RNA extraction, and then synthesis of cDNA with NovoScript plus all-in-one 1st stand cDNA synthesis supermix (Novoprotein, China). Mix the solution to be tested using the SYBR Green real-time PCR mix, and an instrument LightCycler480 RT-PCR system (Roche, Swiss) was used for measurement. *ClCAC* (Gene ID: *Cla97C09G174930*) was used as the reference gene. The original data obtained are calculated as the relative expression level by the 2−ΔΔ^CT^ method (Livak and Schmittgen, 2001).

### Functional analysis of candidate genes

The construction of systematic evolution process: Amino acid sequence of the target gene was obtained from the watermelon reference genome 97103 v2. NCBI BLASTp (https://blast.ncbi.nlm.nih.gov/Blast.cgi) was used to obtain homologous genes of the target gene, database selection ‘UniProtKB/Swiss-Prot (Swissprot)’, and then download the full-length sequence of amino acids for further analysis. MEGE7 (7.0.26) was used for sequence alignment and phylogenetic tree construction, ClustalW was used for amino acid sequence alignment, and Construct/Test neighbor-joining Tree was used for phylogenetic tree construction. The conservative domain was predicted by NCBI Conserved Domains: https://www.ncbi.nlm.nih.gov/Structure/cdd/docs/cdd_search.html; the transmembrane structure analysis was predicted through an online website: TMHMM: http://www.cbs.dtu.dk/services/TMHMM/.

## Results

### Natural variation and correlation analysis of rind thickness and single fruit weight

**Figure 1:**
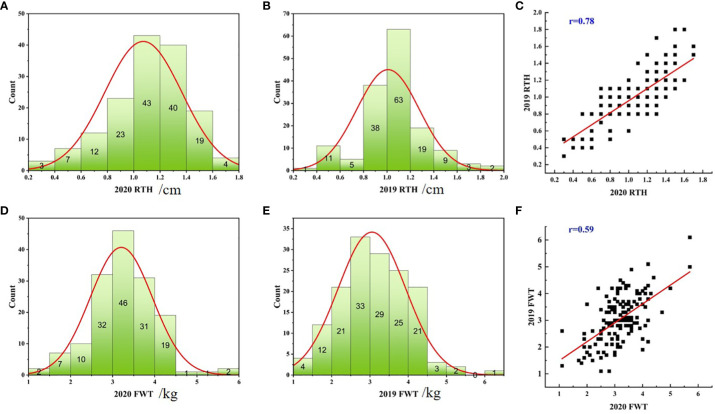
Distribution histograms and correlation graphs of rind thickness and single fruit weight of 151 watermelon accessions in 2019 and 2020. Statistical histogram of the rind thickness in 2020 **(A)**, the rind thickness in 2019 **(B)**, the single fruit weight in 2020 **(D)** and the single fruit weight in 2019 **(E)**, respectively. Correlation diagram of two-year data of rind thickness **(C)** and single fruit weight **(F)**, respectively.

In 2019 and 2020, the coefficient of variation of rind thickness in 151 watermelon accessions was 26.17% and 27.20%, respectively, while the coefficient of variation of single fruit weight was 28.01% and 23.02%, which suggests that these cultivated cultivars had abundant variations in these two agronomic traits. In the process of agricultural production, 0.6cm is an important reference to determine whether watermelon has a thicker rind, statistical analysis showed that there were 14 and 15 accessions with a rind thickness of less than 0.6 cm, 128 and 132 accessions between 0.6-1.6 cm, and 9 and 4 accessions of more than 1.6 cm in two years, respectively. For single fruit weight, there were 19 and 11 accessions with less than 2 kg, 111 and 122 accessions of 2-4 kg, meanwhile, 21 and 18 accessions with more than 4 kg, respectively (**Table S1**). At the same time, most of the kurtosis and skewness of rind thickness and single fruit weight was less than 1 in two years (**Table 1**) and combined with the statistical histogram (**Figures 1A, B**, **Figures 1D, E**), it was concluded that these two important agronomic traits were basically in line with the characteristics of normal distribution in the population. Correlation analysis showed that epidermal thickness had a significant positive relationship in both years (**Figure 1C**), as did single fruit weight (**Figure 1F**), implying that phenotypic differences may be regulated by both genes and the environment, and were the relatively stable quantitative traits.

**Table 1:** Statistical analysis of rind thickness and single fruit weight of 151 watermelon accessions in 2019 and 2020.

Trait	Mean	SD	CV/%	Kurtosis	Skewness
2020 RTH	1.07	0.29	0.27	0.06	-0.54
2019 RTH	1.01	0.26	0.26	0.98	0.08
2020 FWT	3.21	0.74	0.23	1.15	0.13
2019 FWT	3.06	0.86	0.28	0.05	0.34

To explore the relationship between rind thickness and single fruit weight, a correlation analysis between the two traits was performed. The results indicate that there was a positive correlation between rind thickness and single fruit weight, and the correlation values of the two traits were 0.51 and 0.47 in 2019 and 2020, respectively (**Figure S1**). Notably, when the rind thickness was less than 0.6, the average single fruit weight was 2.09 kg (SD=0.53) and 2.40 kg (SD=0.59), respectively; while when the rind thickness was greater than or equal to 0.6, the fruit weigh was 3.16 kg (SD=0.82) and 3.29 kg (SD=0.70). This difference indicates that there was a positive correlation between rind thickness and single fruit weight, especially for the watermelon with thinner rind thickness, which tends to have smaller fruits.

### GWAS and significant SNP acquisition of target traits

**Figure 2:**
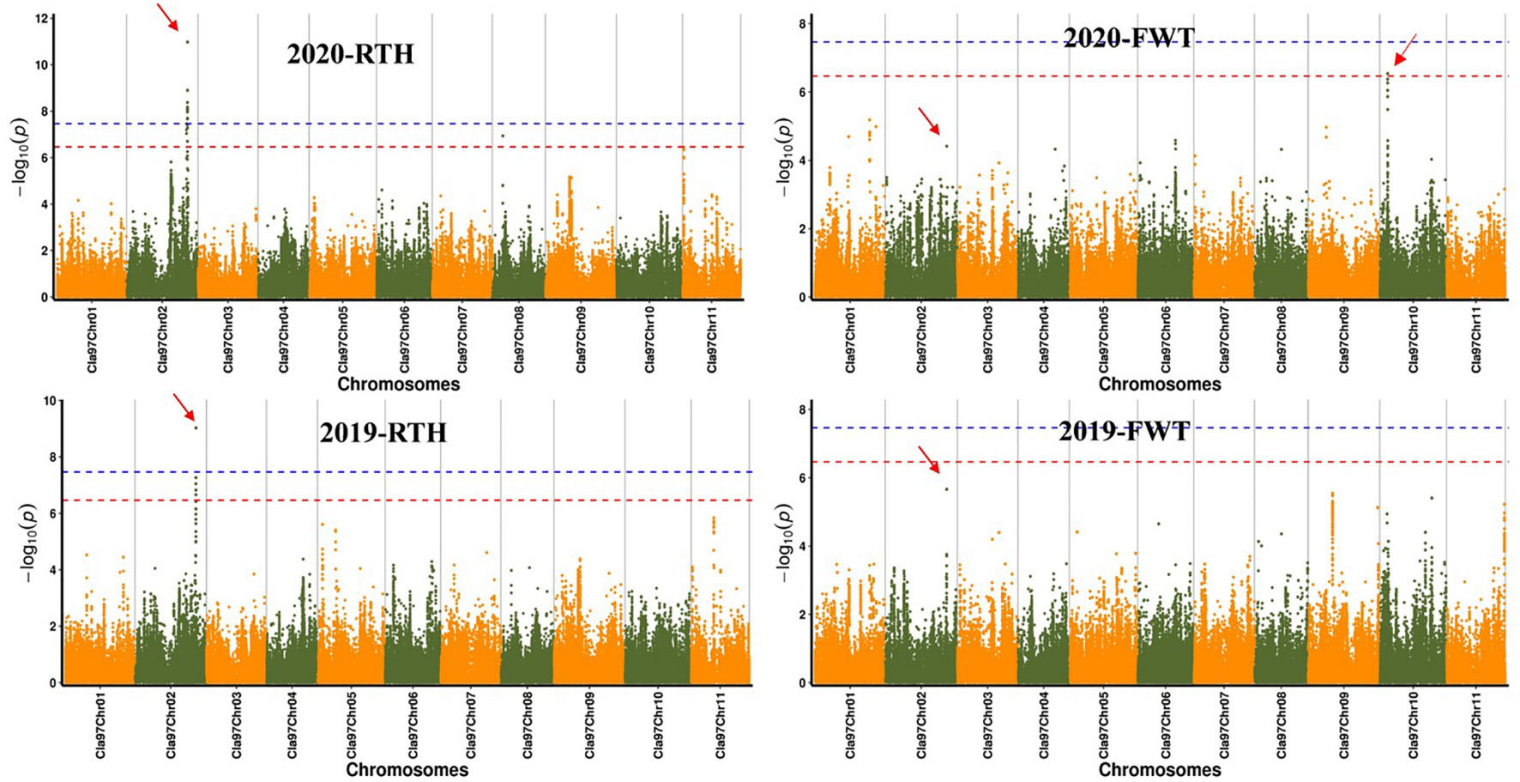
Manhattan plot of watermelon rind thickness and single fruit weight by GWAS in 2019 and 2020. The abscissa represents the chromosome, the ordinate represents -log_10_ (*p*-value), and the red and blue line segments parallel to the coordinate axis represent the two threshold lines, SNPs over red represent highly significant positive associations with traits, SNPs over blue are significantly associated with traits, respectively.

Significant SNPs were obtained by GWAS for rind thickness and single fruit weight in 2019 and 2020 (**Figure 2**), and the QQ plot indicates the reliability of the GWAS analytical model (**Figure S2**). The analysis by the FAST-LMM model of rind thickness found that there were 5 and 16 SNPs that exceeded the threshold line in two years (**Table S2**), and five same significant SNPs (S2: 32344170, S2: 32321308, S2: 32304738, S2: 32328501, S2: 32311192) on chromosome 2 were located on chromosome 2 in 2019 and 2020, and these loci were within the range of 50 kb. Besides, S8:5576295 was a significant SNP of rind thickness on chromosome 8 in 2020, and S10:4123404 was a significant SNP of single fruit weight on chromosome 10. Interestingly, S2: 32344170, which was significantly associated with rind thickness, was also the most significant SNP on chromosome 2 for single fruit weight. It is worth noting that GWAS analysis by EMMAX and LM has relatively similar positioning results with FAST-LMM, which further explains the reliability of our positioning results (**Figure S3**).

### The acquisition of candidate interval and candidate gene

**Figure 3:**
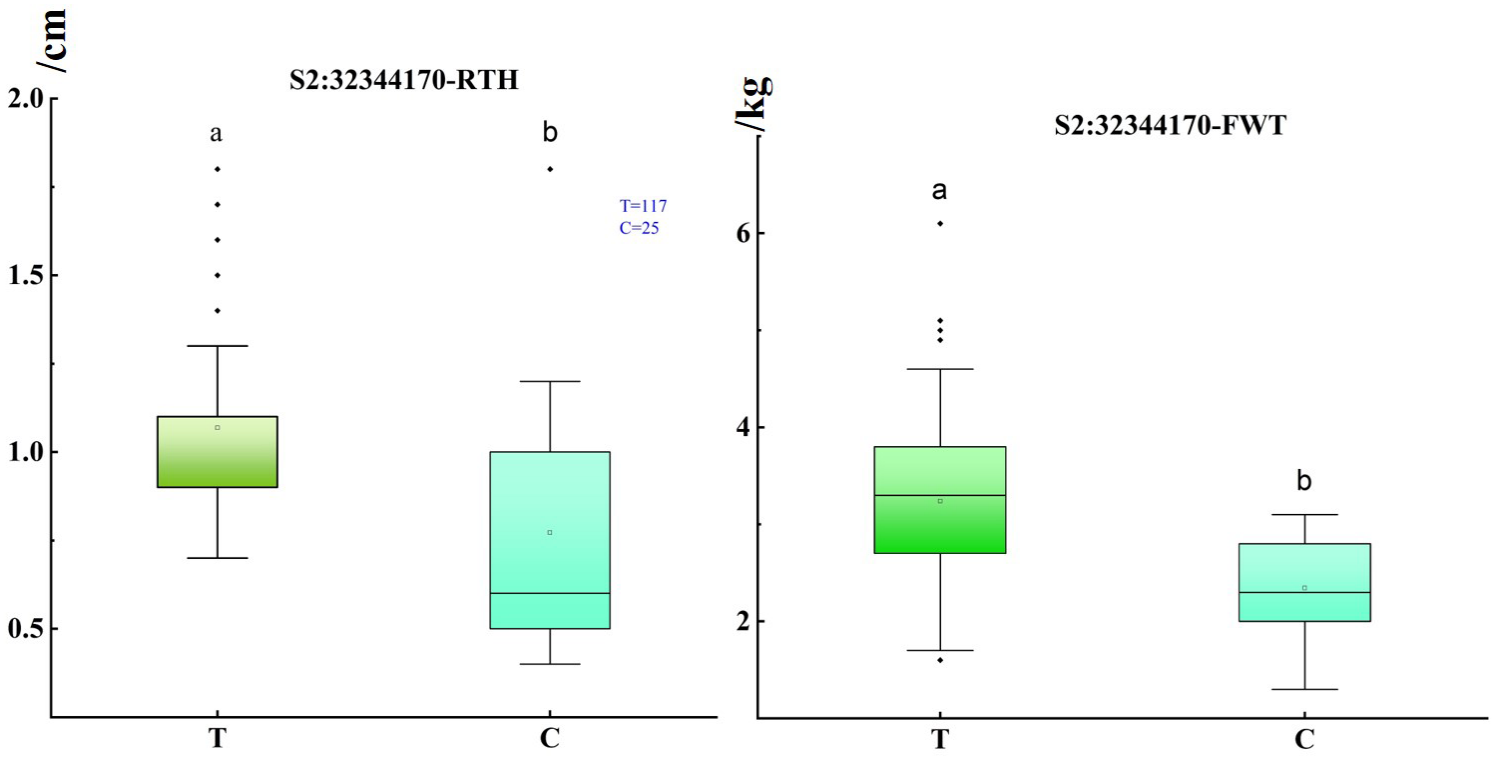
Box plot of single fruit weight and rind thickness under different bases of S2: 32344170. The abscissa represents the alleles with a mutation at the locus, the reference base near the origin, the other one was the key mutation locus, and the ordinate represents the rind thickness and fruit weight, respectively. Significance level: 0.05, a, and b represented significant differences in rind thickness under different alleles. The hollow origin in the boxplot represents the mean value.

Notably, through the above analysis, we found that significant SNP loci were within a candidate interval of about 50 kb. Interestingly, S2: 32304738 and S2: 32311192 were located on the promoter and intron of *Cla97C02G044120*, respectively, and S2: 32321308 and S2: 32328501 were located on the intron and exon of *Cla97C02G044130*, respectively, and S2: 32344170 was the most significant SNP. To obtain allele information of significant SNP based on resequencing data, it is worth noting that when these alleles were in mutant alleles, they have thinner rind thickness and smaller fruit, and this difference was more obvious at S2: 32344170 (**Figure 3**). Moreover, it was found that when the rind thickness was less than 0.6 cm, all the alleles correspond to the mutant alleles (**Table S1**). These results further illustrate the close correlation between significant SNP with target traits on chromosome 2, and S2: 32344170 could be used as potential molecular markers for marker-assisted breeding of target traits. Furthermore, the correlation analysis of regions and genes near the most significant locus S2: 32344170 was analyzed, and the 100 kb upstream and downstream intervals of this site were defined as candidate intervals for further analysis. LD block analysis found that SNP in the candidate interval had a relatively good correlation coefficient (**Figure S4**), indicating that there may be a linkage imbalance in the candidate interval. Interestingly, marker NW0249226 in the previous QTL *Qrth2-1* for rind thickness was only about 50 kb away from S2: 32344170.

A total of 21 genes were obtained in the candidate interval (**Table 2**). GO annotation analysis found that in the molecular_function term, more genes were enriched in GTP binding, molecular_function, protein binding, ATP binding, protein kinase activity, and transferase activity; in the cellular_component term, an integral component of membrane, membrane, nucleus, and plasma membrane had more genes were annotated; in biological_process term, more genes were enriched in protein phosphorylation, MAPK cascade, regulation of transcription, DNA-templated phosphorylation had annotated more genes. It is worth noting that protein phosphorylation, DNA-templated phosphorylation, etc. were often considered to be involved in auxin biosynthesis.

**Table 2:** Candidate gene information of rind thickness and single fruit weight on chromosome 2.

Gene	Location	Gene annotation
*Cla97C02G044060*	Cla97Chr02:32246890.32248741 (+)	transcription elongation factor B polypeptide 3-like
*Cla97C02G044070*	Cla97Chr02:32252262.32256301(-)	Citrate synthase
*Cla97C02G044080*	Cla97Chr02:32264788.32269245(-)	Ubiquinone biosynthesis O-methyltransferase, mitochondrial
*Cla97C02G044090*	Cla97Chr02:32278703.32280043(-)	Leucine-rich repeat receptor-like protein kinase family protein
*Cla97C02G044100*	Cla97Chr02:32283295.32286529(-)	Serine/threonine protein phosphatase 7 long form
*Cla97C02G044110*	Cla97Chr02:32290370.32300164(-)	Serine/threonine-protein phosphatase
*Cla97C02G044120*	Cla97Chr02:32305922.32314246 (+)	elongation factor 2
*Cla97C02G044130*	Cla97Chr02:32320831.32328837 (+)	Protein kinase, putative
*Cla97C02G044140*	Cla97Chr02:32332095.32335291 (+)	elongation factor 2
*Cla97C02G044150*	Cla97Chr02:32337553.32338449(-)	HVA22-like protein
*Cla97C02G044160*	Cla97Chr02:32349097.32356436 (+)	MADS box transcription factor AGAMOUS
*Cla97C02G044170*	Cla97Chr02:32360188.32365056(-)	Hexosyltransferase
*Cla97C02G044180*	Cla97Chr02:32382369.32383409 (+)	Secretion-regulating guanine nucleotide exchange factor
*Cla97C02G044190*	Cla97Chr02:32384480.32385393(-)	protodermal factor 1-like
*Cla97C02G044200*	Cla97Chr02:32387278.32388270(-)	peroxisomal and mitochondrial division factor 2
*Cla97C02G044210*	Cla97Chr02:32390210.32394734(-)	Mitogen-activated protein kinase
*Cla97C02G044220*	Cla97Chr02:32411247.32412302(-)	glucan endo-1,3-beta-glucosidase 12-like
*Cla97C02G044230*	Cla97Chr02:32427610.32430022 (+)	WRKY transcription factor 100
*Cla97C02G044240*	Cla97Chr02:32433191.32433619(-)	Unknown protein
*Cla97C02G044250*	Cla97Chr02:32434065.32437213(-)	Receptor-like protein kinase
*Cla97C02G044260*	Cla97Chr02:32437502.32438686 (-)	Unknown protein

### Gene expression analysis of *Cla97C02G044160*

**Figure 4:**
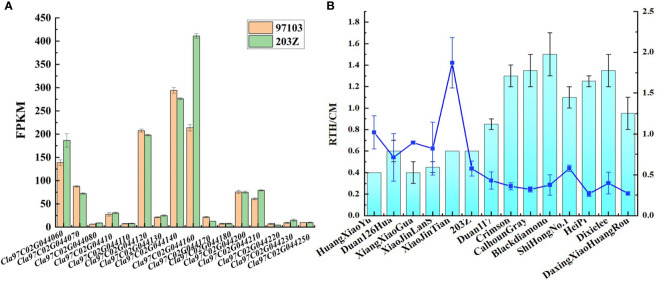
Expression levels of candidate genes for rind thickness and single fruit weight. **(A)** Statistical diagram of gene expression levels among ‘97103’ and ‘203Z’ within the candidate interval. **(B)** Rind thickness and relative gene expression level of *Cla97C02G044160* in 14 watermelon accessions.

Combined with transcriptome data in ‘97103’ and ‘203Z’, the genes in the candidate interval were analyzed. It was found that among the detected genes, only the gene expression level of *Cla97C02G044160* was more than 1.8 times between the two cultivars (**Figure 4A**). The analysis of this gene showed that *Cla97C02G044160* belongs to MADS family, and the typical conserved domains of MADS_MEF2 and K-Box, and transmembrane domain analysis found that K-Box region has a transmembrane region (**Figure S5**). Further combined with phylogenetic tree analysis, it was found that the gene had high homology with the previously reported genes *Osmads34*, *Osmads3*, and *Osmads13* regulating rice organ size (**Figure S6**) (Li et al., 2011; Zhang et al., 2016).

To fully understand the expression of candidate gene *Cla97C02G044160* in the natural population, RNA was extracted from 14 germplasm with different watermelon rind thickness and fruit weight and further used for the qRT-PCR assay. The results showed that the gene expression level was significantly higher when the rind thickness was less than 0.6 cm than when the rind thickness was more than 0.6 cm. Specifically, The gene expression level of *Cla97C02G044160* in ‘HuangXiaoYu’, ‘Duan126Hua’, ‘XiangXiaoGua’, ‘XiaoJinLanS’, ‘XiaoJinTian’, ‘203Z’ was significantly higher than that of ‘Duan117’, ‘Crimson’, ‘CalhounGray’, ‘Blackdiamond’, ‘ShiHongNo.1’, ‘HeiPi’, ‘Dixielee’ and ‘DaxingXiaoHuangRou’ (**Figure 4B**). It should be noted that this trend of gene expression was significantly opposite to that of rind thickness, that is, the gene expression level of *Cla97C02G044160* in rind was higher in watermelon with thinner rind. These results suggest that *Cla97C02G044160* maybe was a key candidate gene for regulating watermelon rind thickness and single fruit weight.

### Transcriptome analysis of cultivars with different phenotypes

The genes whose expression was more than 1.8 times different between ‘97103’ and ‘203Z’ were counted. A total of 12 918 genes were screened for genes with an average content greater than 5. Furthermore, a total of 2117 genes were obtained by screening differentially expressed genes between the two cultivars. These 2117 genes were mainly divided into two categories, one has a higher gene expression in ‘97103’, there are 1241 genes, and the second group had higher gene expression in ‘203Z’, with 876 genes.

**Figure 5:**
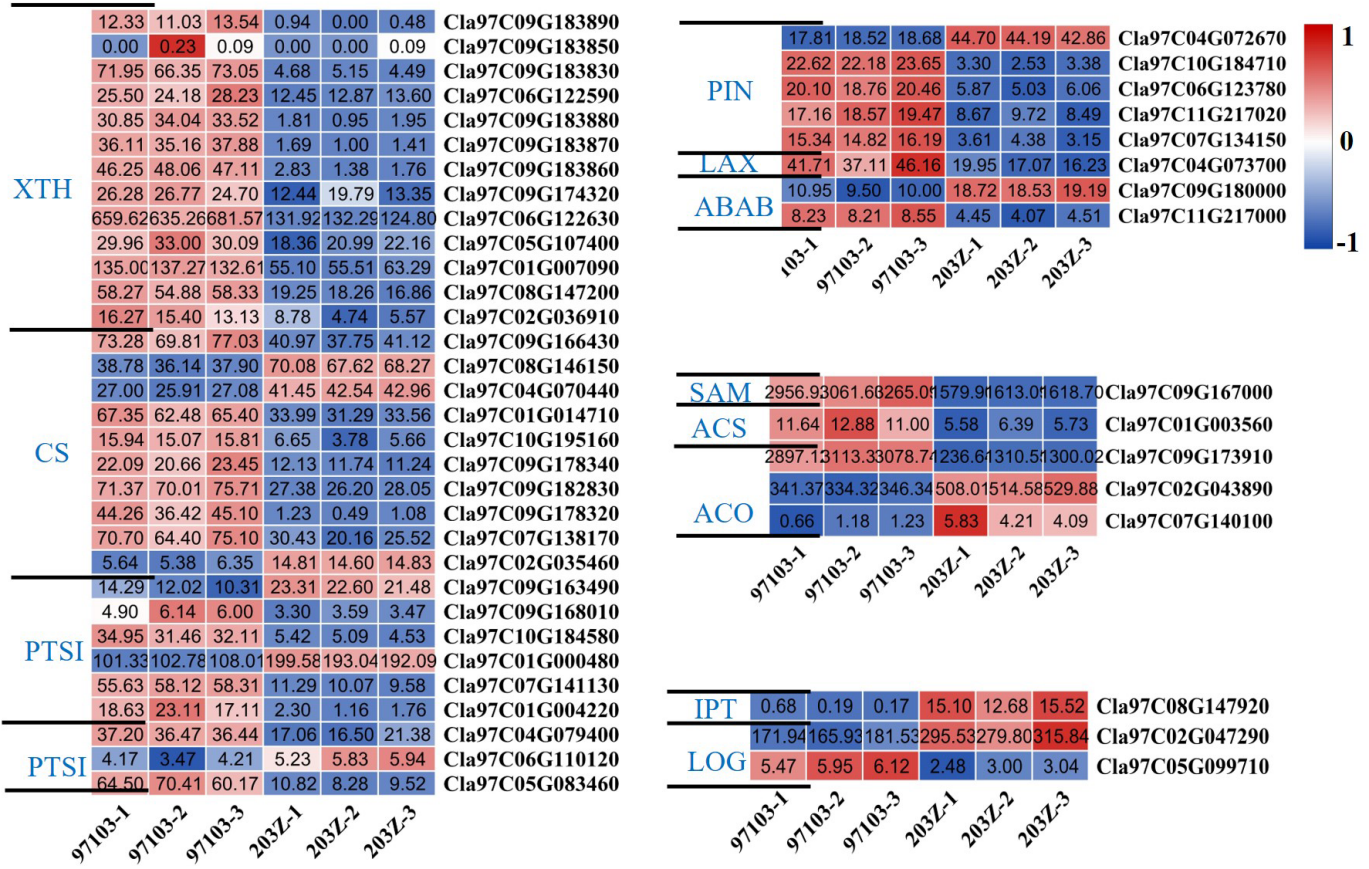
Heat map of differentially expressed genes related to seed expansion or hormone synthesis (auxin, ethylene, cytokinin). The darker the red color in the color scale, the higher the relative expression of the gene in the sample; the darker the blue color, the lower the expression of the gene in the sample. The value in the box represents the FPKM value in the sample. XTH, Xyloglucan endotransglucosylase/hydrolase; CS, Cellulose synthase; PTS, Pectinesterase; PTSI, Pectinesterase inhibitor; PIN, Auxin efflux carrier component; LAX, Auxin transporter-like protein; ABAB, ABC transporter B family member; SAM, S-adenosylmethionine; ACS, 1-aminocyclopropane-1-carboxylate synthase family protein; ACO, 1-aminocyclopropane-1-carboxylate oxidase 1; LOG, Cytokinin riboside 5’-monophosphate phosphoribohydrolase; IPT, Adenylate isopentenyltransferase.

Among these genes, the statistics of genes related to organ expansion revealed that the expression levels of xyloglucan endotransglucosylase/hydrolase, cellulose synthase, pectinesterase, and pectinesterase inhibitor were higher in the watermelon ‘97103’ with thicker rind thickness and heavier fruit; Most of the key genes auxin efflux carrier component, auxin transporter-like protein, ABC transporter B family member related to auxin synthesis had relatively higher gene expression levels in ‘97103’; as well as ethylene synthesis-related genes S-adenosylmethionine, 1-aminocyclopropane-1-carboxylate synthase family protein, 1-aminocyclopropane-1-carboxylate oxidase 1, most of them have higher gene expression levels in ‘97103’; while the cytokinin-related genes cytokinin riboside 5’-monophosphate phosphoribohydrolase, adenylate isopentenyltransferase have higher content in ‘203Z’ (**Figure 5**). Combined with the GO annotation information in the candidate interval based on GWAS, it is speculated that the genes related to the regulation of auxin synthesis were likely to regulate target trait. Combined with the analysis of the expression level of candidate gene *Cla97C02G044160*, these genes with the same or opposite gene expression levels may have a potential regulatory relationship with *Cla97C02G044160*, or may have synergistic effects to make watermelon show different single fruit weight or rind thickness.

## Discussion

Single fruit weight and rind thickness of watermelons are important agronomic traits closely related to domestication and yield. Bahari et al. (2012) screened watermelon cross combinations suitable for commercial cultivation by calculating the general combining ability (GCA) and special combining ability (SCA) of agronomic traits such as rind thickness and fruit weight, to screen out the hybrid combination more in line with the market demand. In the process of domestication, organ expansion is one of the important characteristics, Guo et al. (2019) found the average fruit weight of *C. Colocynthis*, *C. amarus*, *C. mucosospermus*, and *C. lanatus landraces* and cultivars was 0.2, 1.7, 3.7 and 3.4 kg, respectively. However, the artificial selection makes modern cultivated cultivars have a higher degree of domestication, correlation analysis of 151 modern cultivated watermelons showed that the watermelons with thinner rinds tended to have smaller fruits, thus establishing a link between phenotypes. In addition, the abundant variety of watermelon natural populations provides more potential parents for future breeding work.

For gene mapping of quantitative traits, the genetic map is one of the methods to obtain major QTLs for target traits. For instance, Ren et al. (2014) integrated four previously reported genetic maps into a new map of watermelon, and 58 QTLs for 12 traits were mapped into the new map. And interestingly, co-localization of rind thickness (*RTH2-1*) and fruit weight (*FWT2-2*) was found on chromosome 2 and chromosome 5. Moreover, *FWT2-2*, which controls the weight of watermelon fruit, was verified by selective clearance analysis of natural population accessions (Guo et al., 2019). GWAS can quickly obtain molecular markers and candidate genes that are significantly related to traits by detecting genetic variation polymorphisms of multiple individuals in the whole genome and analyzing their association with traits and made important progress in the study of important agronomic traits of watermelon (Dou et al., 2018; Ren et al., 2021). In the present study, 5 significant SNP loci on chromosome 2 were obtained in 2019 and 2020. Interestingly, the highest SNP locus S2: 32344170 was only about 50 kb away from QTL *FWT2-2* and *RTH2-1* (*RTH2-1* was included in QTL *FWT2-2)* (Ren et al., 2014), which regulated watermelon single fruit weight and rind thickness, indicating the reliability of the significant SNPs we obtained. The discovery of molecular markers can be applied to the molecular marker-assisted breeding of crops (Fang et al., 2021; Zhuo et al., 2021), in this study, we found that significant SNPs had significant differences in rind thickness under different bases. For example, under the highest significant SNP, when the rind thickness of cultivated cultivars was less than 0.6 cm, the corresponding base was all C. In summary, these significant SNPs can be used as potential molecular markers in watermelon breeding.

The differences in phenotypic traits are often regulated by genes. Combined with transcriptome data, we obtained the possible candidate gene *Cla97C02G044160* (a MADS family gene) in the candidate interval. In previous reports, genes in the MADS family can negatively regulate the size of tissues and organs by regulating hormones, etc. For instance, *SEEDSTICK* regulates cytokinin levels and other genes to control the fruit size of *Arabidopsis* (Di Marzo et al., 2020); RNA interference of SLMBP21 lead to longer sepals and improved fruit set in tomato, and maybe a significant correlation with auxin and ethylene (Li et al., 2017). In cucumber fruit, *CsFUL1A* inhibits the expression of auxin transporters *PIN-FORMED1* (PIN1) and *PIN7* and then resulting in decreases in auxin (Zhao et al., 2019). Zheng et al. (2020) found that two MADS-box transcription factors, *VCM1* and *VCM2*, play an important role in tree thickening. The discovery that the MADS family gene *Cla97C02G044160* could negatively regulate the size of tissues and organs was also consistent with this study, that is, the expression level of this gene in watermelon ‘203Z’ with lighter fruit weight and the thinner rind was significantly higher than ‘97103’. In addition, combined with transcriptome data, it was further found that key genes related to organ expansion and auxin and ethylene had a higher accumulation amount in ‘97103’, and our study further improved the transcriptional regulation basis of watermelon rind thickness and single fruit weight.

## Conclusions

In the current study, we identified natural variations and correlations between rind thickness and single fruit weight in 151 watermelon accessions, and the significant SNP loci obtained by GWAS may serve as potential molecular markers to participate in breeding. Further combined with transcriptome data and bioinformatics analysis, it was found that *Cla97C02G044160* may be involved in the phenotypic changes of the target trait. Our research provides rich data resources for the selection of new cultivars and molecular marker-assisted breeding.

## Data availability statement

The data presented in the study are deposited in the NCBI repository, accession numbers PRJNA641178 and PRJNA905076, all data is publicly available and can be used with the permission of the authors.

## Author contributions

WL, NH, and CG designed this experiment. WL, XL, NH, and HZ were mainly responsible for the collection of watermelon accessions and plants management. WL and CG were mainly responsible for the statistics of phenotypic data. CG was mainly responsible for the data collation and analysis, and completed the writing of the original manuscript. WL, NH, and MA were mainly responsible for the manuscript verification. All the authors agreed to the submission of the manuscript. All authors contributed to the article and approved the submitted version.
