# Supplementary material for: Genome-wide association study provides genetic insights into natural variation in watermelon rind thickness and single fruit weight
Source: Front Plant Sci. 2022 Dec 6;13:1074145. doi: 10.3389/fpls.2022.1074145 (PMC9763438; doi:10.3389/fpls.2022.1074145)
Supplement: Supplementary file 1 [file DataSheet_1.docx]

**Supplementary figures 1-6**


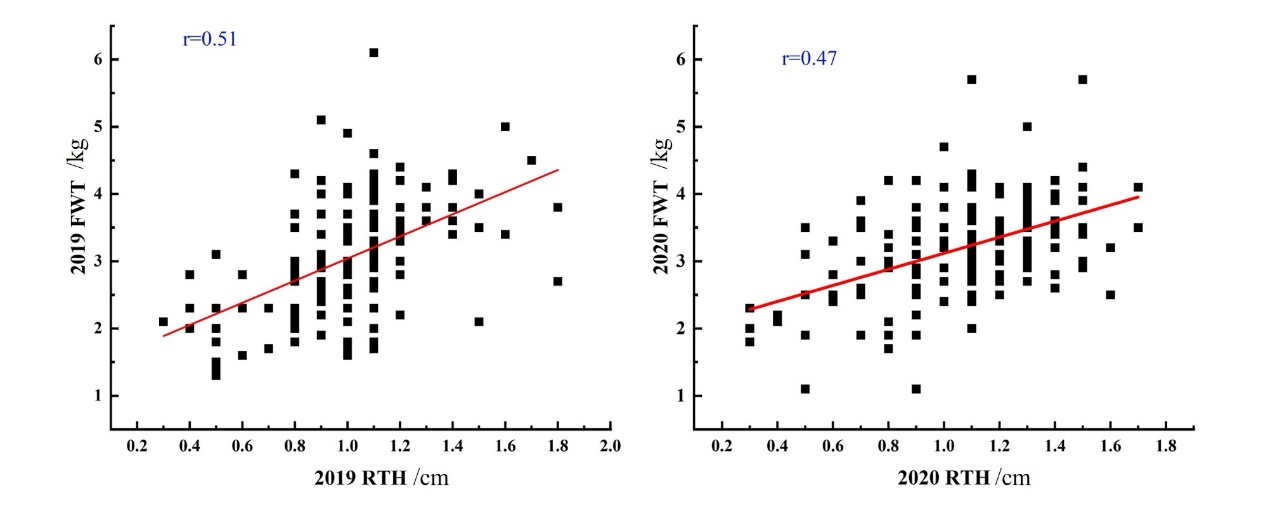


**Fig. S1** Correlation analysis of rind thickness (RTH) and fruit weight (FWT) in 2019 and 2020.


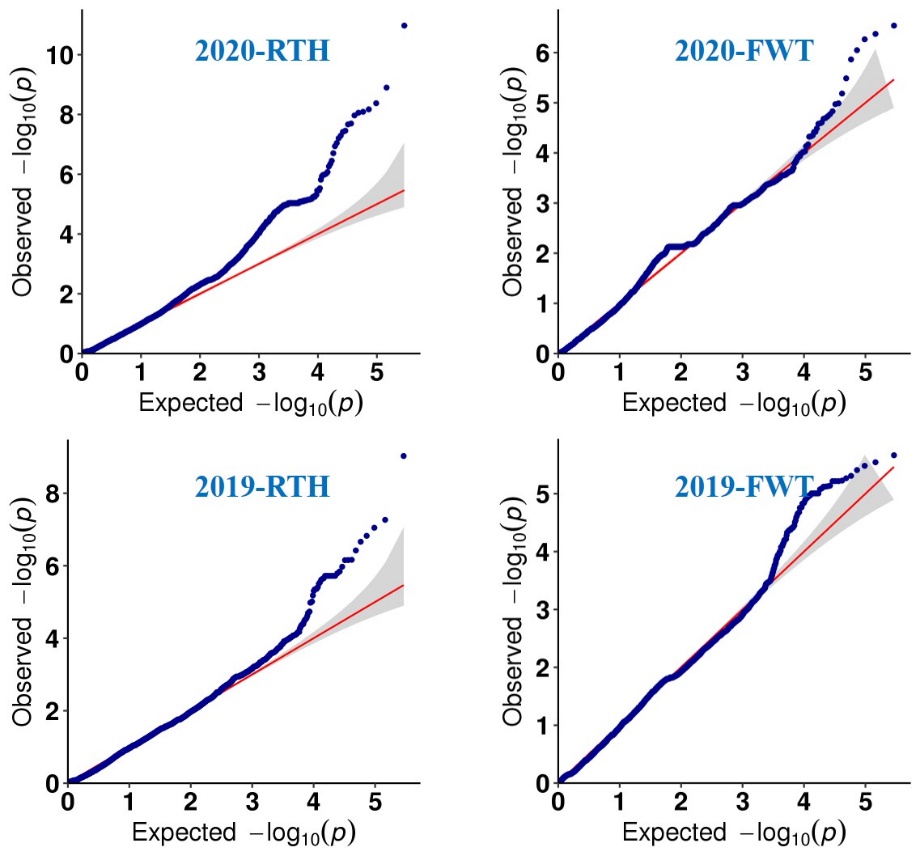


**Fig. S2** QQ plot of rind thickness and fruit weight in 2019 and 2020.


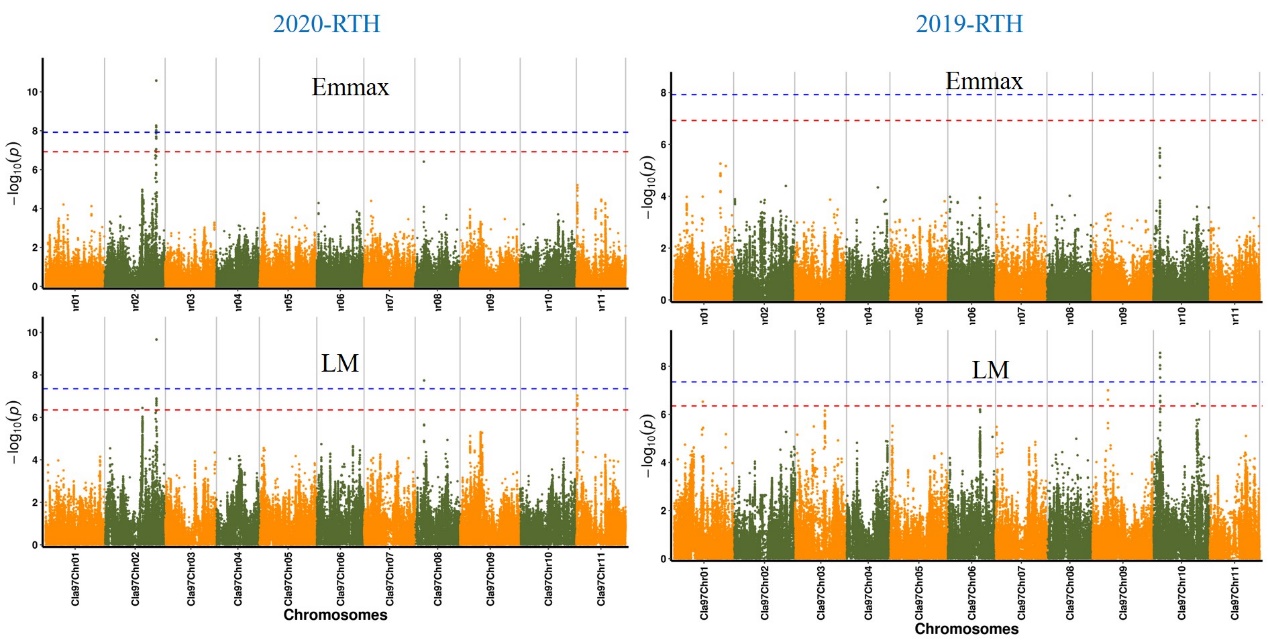


**Fig. S3**  GWAS of single fruit weight and peel thickness in 2020 using EMMAX and LM models.


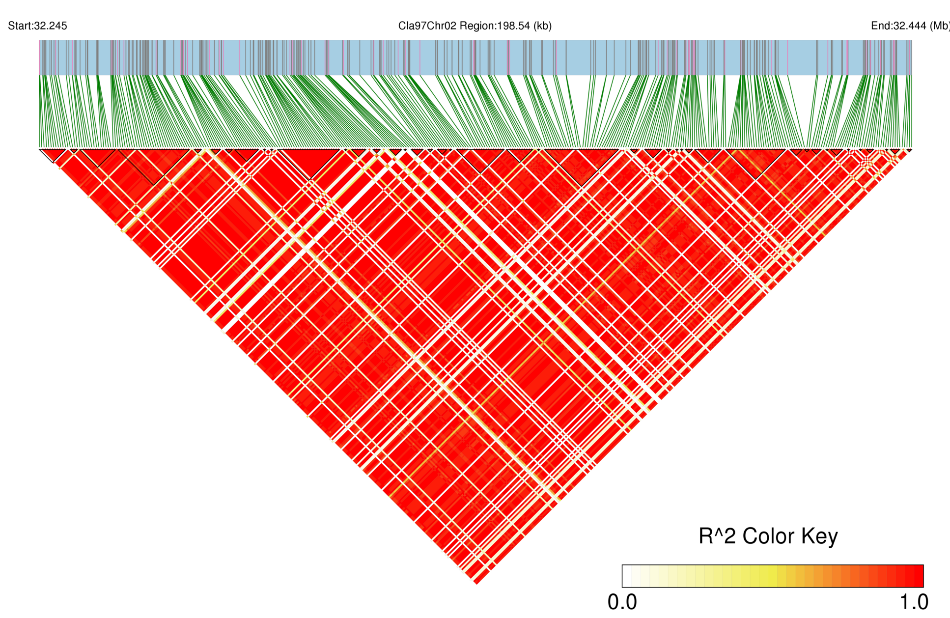


**Fig. S4** LD block analysis of all SNP data in candidate regions at about upstream and downstream 100 kb of significant SNP locus S2: 32344170.


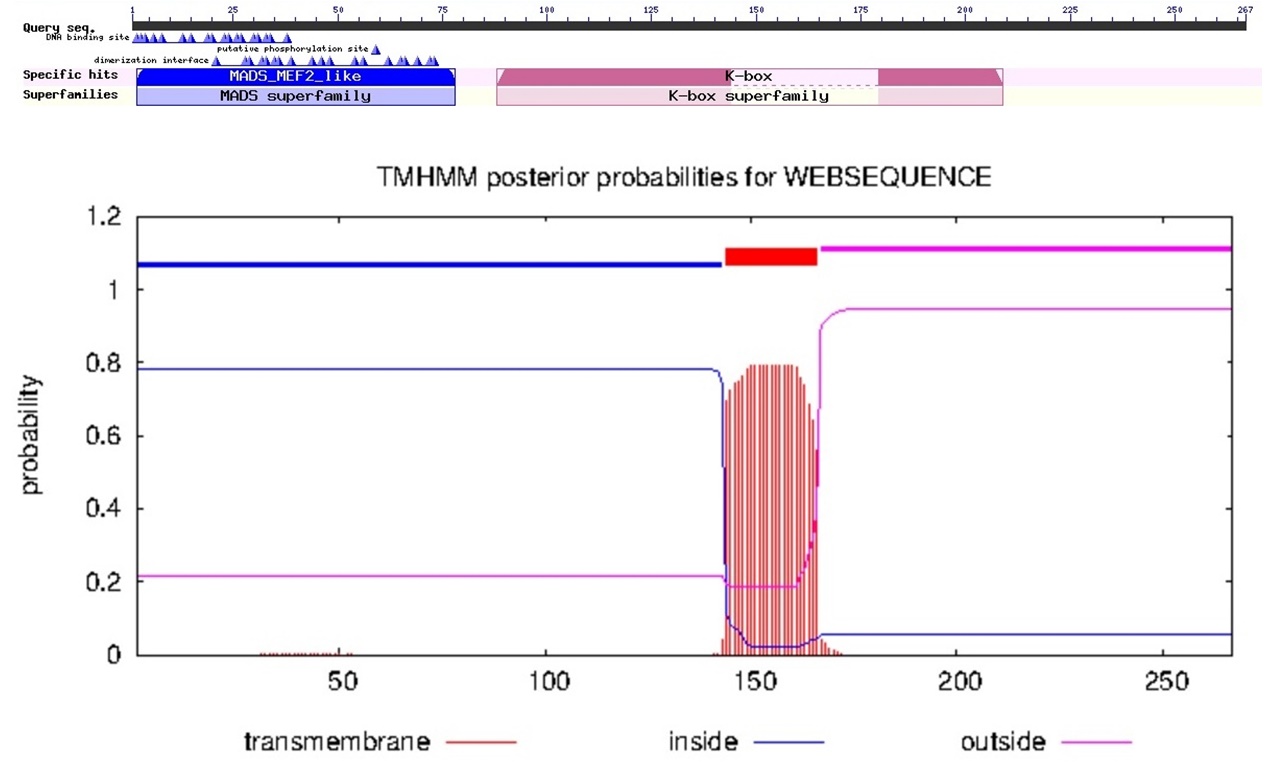


**Fig. S5** Prediction of conservative and transmembrane domains of *Cla97C02G044160.*


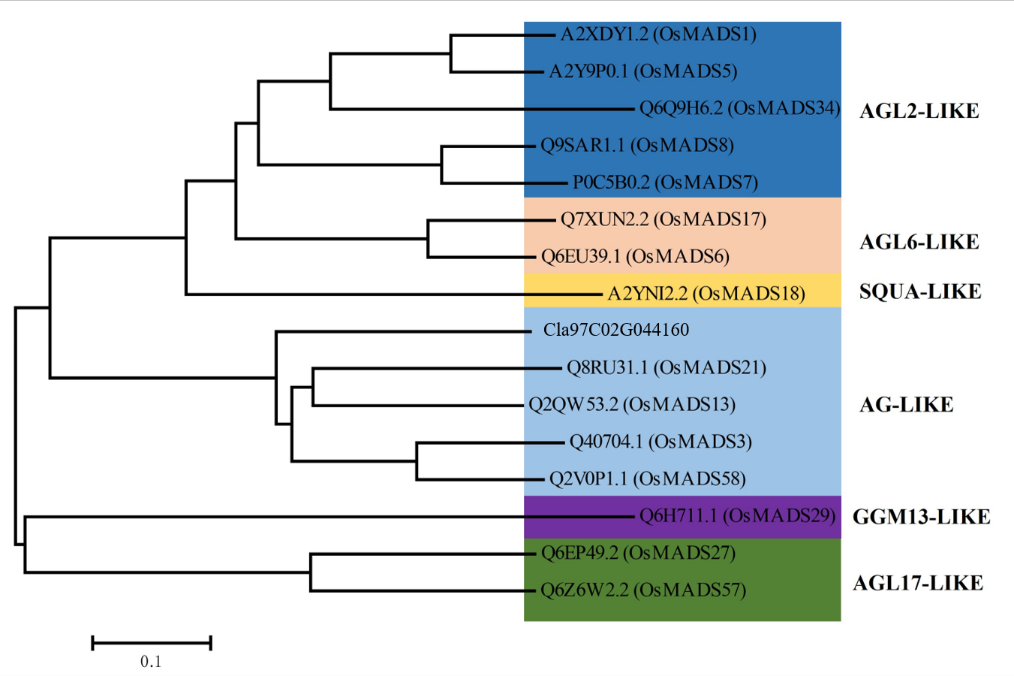


**Fig. S6** Phylogenetic tree analysis of 15 MADS family genes in rice and *Cla97C02G044160*. Genes with different colors represent different clades.
