# Supplementary material for: Genome-wide association study provides genetic insights into natural variation in watermelon rind thickness and single fruit weight
Source: Front Plant Sci. 2022 Dec 6;13:1074145. doi: 10.3389/fpls.2022.1074145 (PMC9763438; doi:10.3389/fpls.2022.1074145)
Supplement: Supplementary file 2 [file Table_1.docx]

**Table S1Phenotypic data of peel thickness and fruit weight, and SNP information of S3 in 151 watermelon accessions**

| **Accession** | **ID** | **2020 RTH** | **2019 RTH** | **2020 FWT** | **2019 FWT** | **S2:32344170** |
| --- | --- | --- | --- | --- | --- | --- |
| WM152 | QiaErTaWuZi | 0.8 | 1.1 | 1.7 | 1.7 | T |
| WM287 | MoonAndStar | 1.1 | 1.1 | 4.1 | 4.2 | T |
| WM292 | Duan117 | 0.8 | 0.9 | 1.9 | 2.5 |  |
| WM361 | XiHua | 0.6 | 0.8 | 2.5 | 2.3 | C |
| R38 | HXD | 0.8 | 0.9 | 2.9 | 3.4 |  |
| WM363 | 98002 | 1.2 | 1 | 3 | 3.4 | T |
| WM364 | 92002 | 1.1 | 0.9 | 3.6 | 3 | T |
| WM367 | LCHF | 1.4 | 1 | 4 | 2.3 | T |
| WM369 | HM | 1.1 | 0.9 | 4.3 | 4 | T |
| WM272 | XiangXiaoGua | 0.3 | 0.5 | 1.8 | 1.4 | C |
| WM274 | HuangXiaoYu | 0.4 | 0.4 | 2.1 | 2 | C |
| WM380 | DaxingXiaoHuangRou | 1.1 | 0.8 | 3.2 | 2.7 | T |
| WM383 | XiaoJinLanS | 0.5 | 0.4 | 3.5 | 2.8 | C |
| WM384 | TeXiaoFengS | 1.1 | 1 | 3.3 | 3 | T |
| WM390 | YU18 | 0.3 | 0.5 | 2 | 1.8 | C |
| WM246 | HuaDongNo.26 | 0.9 | 0.9 | 2.8 | 3.7 | T |
| WM206 | NingXiaHongZiGua | 1.1 | 1 | 2.4 | 1.8 | C |
| R33 | LiuTiaoQing | 1.5 | 1.6 | 5.7 | 5 | T |
| WM284 | FuZhouGua | 1.2 | 1.1 | 4 | 3.6 | T |
| WM285 | MaLingGua | 1.2 | 1.2 | 3.4 | 3.3 | T |
| WM373 | TangPaoDan | 0.3 | 0.3 | 2.3 | 2.1 |  |
| WM155 | LaMaGua | 1.3 | 1.1 | 4 | 2.3 | T |
| WM157 | LiPi | 1.2 | 0.8 | 3.4 | 2.8 | T |
| R15 | HeTaoPi | 1.1 | 0.9 | 3.6 | 2.7 | T |
| WM176 | QingBaoJin | 1.3 | 1.1 | 3 | 1.8 | T |
| WM178 | ShanxiBai | 0.8 | 0.7 | 3 | 1.7 | T |
| WM212 | QiTouHuang | 1.1 | 1.1 | 4.1 | 4 | T |
| R14 | TaoJian | 1 | 1.2 | 3.3 | 3.3 |  |
| WM179 | ZaoMiAi | 0.9 | 1 | 2.2 | 2.6 | C |
| R18 | DongGua | 0.8 | 1.1 | 3.4 | 3.1 | T |
| R20 | HeiBengJin | 0.7 | 0.9 | 2.6 | 3 | T |
| WM213 | JinBaoYin | 1.3 | 1.4 | 3.9 | 3.8 | T |
| WM359 | ChengLan | 1.1 | 1 | 3.7 | 3.7 | T |
| WM360 | W1 | 1.2 | 1 | 3 | 3 | T |
| WM216 | ZhengzhouNo.1 | 0.7 | 1 | 3.6 | 4.9 | T |
| WM198 | BaiGuaZi | 1.6 | 1.2 | 2.5 | 3.4 | T |
| WM204 | HongGuaZi | 0.9 | 1.1 | 1.1 | 2.6 | C |
| WM343 | DaHongTian | 1.7 | 1.6 | 3.5 | 3.4 | T |
| WM148 | KaLaQiPa | 1.5 | 1.4 | 3.4 | 3.6 | T |
| WM149 | DaHongZi | 1.2 | 1.1 | 3.5 | 4.3 | T |
| WM150 | ALaKeZiWai | 0.8 | 1 | 1.9 | 2.5 | T |
| WM159 | NingXianXiGua | 1 | 0.8 | 3.5 | 3.7 | T |
| WM163 | KuiKeTaWuZi | 1.6 | 1.8 | 3.2 | 2.7 | C |
| WM171 | JiZhuaGua | 0.6 | 0.4 | 2.4 | 2.3 | C |
| WM175 | HuaLing | 0.9 | 0.8 | 4.2 | 4.3 | T |
| R17 | TaCheHong | 1.1 | 1.4 | 2.5 | 3.4 |  |
| R29 | ChangHui | 0.9 | 0.9 | 2.8 | 2.5 | T |
| WM85 | TOMATOSEED | 1.5 | 1.8 | 3 | 3.8 | T |
| WM342 | Hongdneg | 1.2 | 1.5 | 3.1 | 2.1 | T |
| WM291 | Duan128Huang | 0.9 | 1 | 1.9 | 1.6 | T |
| WM293 | XBM | 1.2 | 1.1 | 3.1 | 4 | T |
| R34 | Crimson | 1.4 | 1.2 | 4.2 | 3.8 | T |
| WM307 | ZhongYuNo.10 | 1 | 1 | 4.7 | 3.3 | T |
| WM325 | Jin5Fu | 1.2 | 1.3 | 3.4 | 3.6 | T |
| WM328 | YingPiNo.2 | 1.1 | 0.8 | 2.9 | 2.3 | T |
| WM266 | LianXiao-5 | 1.2 | 1.1 | 3.6 | 3.5 | T |
| WM188 | SiLaFuPaiKa | 1.4 | 1.2 | 3.5 | 3 | T |
| WM191 | MeiHao | 1 | 0.8 | 3.8 | 2.9 | C |
| WM222 | XingchengHong | 1.4 | 1.2 | 4.2 | 2.2 | T |
| R25 | CalhounGray | 1.5 | 1.2 | 2.9 | 4.2 | T |
| R26 | Blackdiamond | 1.3 | 1.7 | 3.2 | 4.5 | T |
| WM238 | Dixielee | 1.5 | 1.2 | 3.5 | 3.6 | T |
| WM320 | Mimei | 1 | 1.1 | 2.7 | 3.9 | T |
| R27 | WuChaZao | 1 | 0.9 | 2.4 | 4.2 | T |
| WM242 | 27150 | 1.1 | 1.4 | 3.4 | 4.2 | T |
| WM244 | XiaoZiNo.4 | 1.1 | 1.1 | 2 | 2.6 | C |
| WM301 | SanYi | 1.3 | 1.2 | 5 | 4.2 | T |
| WM335 | ShouJinTiao | 1.2 | 1 | 4 | 4.1 | T |
| WM338 | ZhouZhiHong | 1.4 | 1.1 | 3.6 | 2.6 | T |
| WM169 | TaiLiHong | 0.8 | 0.8 | 3.2 | 2.8 | T |
| WM172 | SuXianXiaoZi | 0.8 | 0.7 | 2.1 | 1.7 | T |
| WM182 | YanShiNo.1 | 1 | 0.8 | 4.1 | 3.5 | T |
| WM211 | BinLangPi | 0.4 | 0.5 | 2.2 | 1.5 | C |
| WM220 | 2000B57Ban | 0.7 | 0.9 | 3.5 | 2.5 |  |
| R13 | TuBaiPiXiGua | 1.5 | 1.1 | 4.4 | 4.6 | T |
| WM350 | HuTuBiZaoShu1 | 0.5 | 0.5 | 3.1 | 2 | C |
| WM153 | LanzhouHuaPi | 1.1 | 1.1 | 4.2 | 3 | C |
| WM158 | TouXinHong | 1.1 | 1.1 | 3 | 3.5 | T |
| WM165 | MaSiHe | 1.1 | 1.1 | 3.8 | 3.1 | C |
| R16 | FuYangYiHao | 1.3 | 1.3 | 3.1 | 4.1 | T |
| WM168 | HeiPi | 1.3 | 1.2 | 3.3 | 4.4 | T |
| WM174 | WeiShi | 1.5 | 1.5 | 3.9 | 4 | T |
| WM185 | XiaoMaZi | 1.3 | 1.2 | 2.9 | 3.5 | T |
| WM189 | MeiLi | 1.1 | 0.8 | 3.1 | 2.8 | T |
| WM207 | GuangZhouHuaPi | 1.3 | 0.9 | 3.7 | 2.9 | T |
| WM208 | DingXinHong | 1.7 | 1.5 | 4.1 | 4 | T |
| WM251 | XiangJiuShan | 1 | 0.8 | 3.3 | 2.2 | T |
| WM252 | Xiaoxigua-4 | 1.4 | 1.1 | 2.6 | 1.8 | T |
| WM253 | WuChaXiGua | 0.6 | 0.5 | 3.3 | 3.1 | C |
| WM256 | ZhuXiaoHeiXiaoZi | 0.9 | 0.9 | 3.3 | 2.2 | T |
| WM258 | ABXi | 1.1 | 0.9 | 2.8 | 2.5 | T |
| WM259 | QiongLu | 1.1 | 1 | 2.9 | 2.8 | T |
| WM260 | LuoFeiLin | 1.4 | 1.5 | 2.8 | 3.5 | T |
| WM262 | HongNo.1 | 1.1 | 1 | 3.4 | 3 | C |
| WM264 | XuDaHe | 1.2 | 1.1 | 3.1 | 3 | T |
| R30 | MeiGuoDuanMan | 1.2 | 1 | 2.8 | 2.1 | T |
| R31 | XinDaHe | 1.2 | 0.9 | 2.5 | 2.6 | T |
| R32 | PI161375 | 1.3 | 1 | 2.7 | 1.7 | T |
| WM278 | NongChong | 1.2 | 1 | 3 | 2.5 |  |
| WM288 | Duan125ChangGuo | 0.9 | 1 | 2.5 | 2.5 | T |
| WM289 | Duan126Hua | 0.5 | 0.7 | 1.9 | 2 | C |
| WM294 | Dusx | 1.1 | 1.1 | 3.2 | 3.2 | T |
| WM296 | T1f | 1.4 | 1.2 | 3.2 | 4.2 | T |
| WM300 | ShiHongNo.1 | 1.2 | 1 | 2.8 | 3.3 | T |
| WM303 | ShiHongNo.2 | 1.3 | 0.8 | 3.2 | 3 | T |
| WM305 | XBF | 1.5 | 1.2 | 4.1 | 2.8 | T |
| WM306 | G5F | 1.4 | 1.1 | 3.6 | 3.1 | T |
| WM308 | TaiGuChang | 1.1 | 1 | 3.3 | 3.5 | T |
| WM309 | GuiYinNo.6 | 1 | 1.2 | 3.2 | 3.6 | T |
| WM311 | SBDHei | 1.3 | 1.1 | 4.1 | 3.5 | T |
| WM313 | ChangMiBao | 1.1 | 1.1 | 3.6 | 2.6 | T |
| WM315 | FaHei | 1.2 | 1 | 3.6 | 3.5 | T |
| WM317 | XinXiLan | 1.1 | 1.1 | 3.1 | 3.7 | T |
| WM318 | XinJiangNo.2 | 1.1 | 1.1 | 5.7 | 6.1 | T |
| WM319 | EDNo.2 | 1.2 | 1.2 | 4.1 | 4.2 | T |
| WM321 | XiaoZiMiBao | 1.3 | 1 | 3.8 | 4 | T |
| WM324 | JiangJun | 1.4 | 1.3 | 3.9 | 3.8 | T |
| WM326 | 9904 | 1.1 | 1.1 | 2.7 | 3.3 | T |
| R35 | LSW-194 | 1.4 | 1.4 | 3.4 | 4.3 | T |
| WM330 | XiChunXuan | 0.9 | 0.9 | 3.1 | 2.4 | T |
| WM331 | HongBaoShi | 1.1 | 1 | 3.2 | 3 | T |
| WM334 | Jinzhou204 | 1 | 0.6 | 2.9 | 2.3 | C |
| WM337 | XYXA | 1.3 | 1 | 2.9 | 2.9 | T |
| WM340 | 98A13 | 0.9 | 0.8 | 2.9 | 2.1 | T |
| R36 | MiQiLi | 1.3 | 1.1 | 3 | 3.3 | T |
| WM345 | LvBeiLei | 0.9 | 0.8 | 3.6 | 2.8 | T |
| WM346 | TuoPaKe | 1.2 | 0.9 | 4 | 1.9 | T |
| WM353 | ZhengzhouNo.3 | 1.1 | 0.9 | 3.8 | 2.9 | T |
| R37 | 89xuan9 | 1.3 | 1 | 3.6 | 2.8 | T |
| WM371 | DongFangMeiJia | 1.3 | 1.1 | 3.8 | 3.6 | T |
| WM372 | 790010 | 1.2 | 1.1 | 3.3 | 3.7 | T |
| R22 | ZhengzhouNo.2 | 1.2 | 1.1 | 3.1 | 4.2 | T |
| WM276 | DuanMan | 0.5 | 0.5 | 1.1 | 1.3 | C |
| WM378 | 02d97 | 0.7 | 0.7 | 1.9 | 2.3 | T |
| WM381 | XiuLanS | 0.5 | 0.5 | 2.5 | 2.3 | C |
| WM389 | XiaoHongYu | 1.1 | 0.9 | 2.8 | 2.9 | T |
| WM392 | 203Z | 0.6 | 0.6 | 2.8 | 2.8 | C |
| WM393 | XiaoJinTian | 0.6 | 0.6 | 2.5 | 1.6 | C |
| R19 | XiaoQingPi | 1.1 | 1 | 3.1 | 2.3 |  |
| WM186 | SulianNo.2 | 1.3 | 1.2 | 3.5 | 2.8 | C |
| WM209 | DaXigua | 1.1 | 1.1 | 3.4 | 3.3 | T |
| WM214 | ZhongShiHong | 0.9 | 0.9 | 3.5 | 3.1 | T |
| R21 | ZhongYuNo.3 | 0.9 | 1.1 | 2.6 | 2.7 | T |
| WM225 | 2002D80 | 0.7 | 0.8 | 2.5 | 1.8 | T |
| WM227 | ZhengZhouYCL | 0.7 | 1.1 | 3 | 3 | T |
| WM228 | MoxigeHeiPi | 0.9 | 1.1 | 3.8 | 4.1 | T |
| R24 | Sugarlee | 0.7 | 0.9 | 3.9 | 2.7 | T |
| R28 | ZaoHua | 1.1 | 0.8 | 2.7 | 2.1 |  |
| WM241 | BianliangNo.1 | 1.1 | 1.1 | 3.2 | 2 | T |
| WM243 | SuMiNo.1 | 1.2 | 1.1 | 2.7 | 2.9 | T |
| WM245 | KaiZaNo5.1 | 0.8 | 0.9 | 4.2 | 5.1 | T |

**Table S2: Significant SNP loci information for fruit weight and rind thickness in 2019 and 2020**

| **Trait** | **Locus name** | **Chromosome** | **Locus** | **P_value** |
| --- | --- | --- | --- | --- |
| 2020-RTH | S2:32344170 | Cla97Chr02 | 32344170 | 1.06E-11 |
|  | S2:32328501 | Cla97Chr02 | 32328501 | 1.26E-09 |
|  | S2:32286806 | Cla97Chr02 | 32286806 | 4.21E-09 |
|  | S2:32301548 | Cla97Chr02 | 32301548 | 6.74E-09 |
|  | S2:32311215 | Cla97Chr02 | 32311215 | 8.14E-09 |
|  | S2:32311192 | Cla97Chr02 | 32311192 | 8.76E-09 |
|  | S2:32283703 | Cla97Chr02 | 32283703 | 1.08E-08 |
|  | S2:32352103 | Cla97Chr02 | 32352103 | 2.01E-08 |
|  | S2:32304738 | Cla97Chr02 | 32304738 | 2.15E-08 |
|  | S2:31812052 | Cla97Chr02 | 31812052 | 3.5E-08 |
|  | S2:32321308 | Cla97Chr02 | 32321308 | 3.83E-08 |
|  | S2:32323124 | Cla97Chr02 | 32323124 | 5.17E-08 |
|  | S2:31810348 | Cla97Chr02 | 31810348 | 6.28E-08 |
|  | S2:31811607 | Cla97Chr02 | 31811607 | 9.08E-08 |
|  | S8:5576295 | Cla97Chr08 | 5576295 | 1.15E-07 |
|  | S2:32304592 | Cla97Chr02 | 32304592 | 1.98E-07 |
| 2019-RTH | S2:32344170 | Cla97Chr02 | 32344170 | 9.41E-10 |
|  | S2:32321308 | Cla97Chr02 | 32321308 | 5.43E-08 |
|  | S2:32304738 | Cla97Chr02 | 32304738 | 8.96E-08 |
|  | S2:32328501 | Cla97Chr02 | 32328501 | 1.5E-07 |
|  | S2:32311192 | Cla97Chr02 | 32311192 | 2.18E-07 |
| 2020-FWT | S10:4123404 | Cla97Chr10 | 4123404 | 2.89E-07 |
